# Notes and News

**Published:** 1895-07-20

**Authors:** 


### NOTES AND NEWS.

(Collected and Communicated.)

HOSPITAL MEETINGS, &c.

The Annual Meeting of the Sailors' Orphan Girls' School and Home will be held at the Home, Fitzjohn's Avenue, Hampstead, on July 23rd, at half-past three p.m., Admiral Sir F. Leopold McClintock, K.C.B., F.R.S., in the chair.

Opening of the new front building of the Royal Free Hospital, at one o'clock on Monday, July 22nd, by the Prince and Princess of Wales.

Forty thousand francs have already been subscribed towards erecting a monument to Charcot. Foreigners have contributed largely. The monument is to be erected by his friend Falguière.

The medical staff and lecturers of the Dental Hospital of London will hold a conversazione at the Royal Institute Galleries on Thursday, July 25th, at eight p.m., on the occasion of the distribution of prizes by Sir William MacCormac.

Sir Eric Erichsen will present Sir Joseph Lister with his portrait on the 30th inst., at King's College Hospital. The portrait has been painted by Mr. J. H. Lorimer, A.R.S.A., and is a tribute from Sir Joseph Lister's former colleagues and pupils.

Sir Philip Smyly has been appointed surgeon to the Queen in Ireland, in the place of the late Sir George Porter, Bart. He has acted as physician to the Lord Lieutenant, is surgeon to the Meath Hospital, and was surgeon to various Viceroys of Ireland.

Dr. Vintras, of the French Hospital in London' has received the order of St. Maurice and St. Lazare from the King of Italy, on the occasion of the marriage of the Duke of Aosta, for his services to poor Italian patients.

Monsieur Faure continues to show his interest in the care of the sick. He recently visited the Hotel Dieu in Paris, and, after an inspection of the establishment, presented medals and decorations to various medical men and to ladies occupied in nursing at various institutions.

The Municipal Council and the General Council of Paris have presented Dr. Roux with a gold medal each on behalf of the citizens of Paris.

At the annual banquet of this society, held in March last, various medals were awarded, amongst others the silver medal to Dr. Clement Dukes, M.R.C.S.

The latest created medical knights are Mr. Thornley Stoker, president of the Royal College of Surgeons, Ireland, and Dr. Christopher Nixon, senior physician of the Mater Misericordæ Hospital and professor of medicine in the Catholic University Medical School.

At a meeting held this week, it was resolved the Memorial to Professor Huxley should take the form of a Huxley Scholarship and Medal to be awarded annually at Charing Cross Hospital Medical School, and that, if funds permit, an annual lecture at the Charing Cross Hospital Medical School, dealing with recent advances in science and their bearing upon medicine, should be instituted. The committee appointed to carry out the resolution are Sir Joseph Fayrer, Sir Guyer Hunter, Dr. Watt-Black, Mr. J. Morgan, Mr. Stanley Boyd, Mr. H. Huxley, Dr. Montague Murray, and Mr. H. Waterhouse.

The presentation of the Victoria Cross to Surgeon-Captain Whitchurch, who so gallantly endeavoured to save the life of Captain Baird before Chitral, will be one of the most popular awards in the Chitral honour list. The Medical Corps may be proud that thirteen of their number, beside Surgeon Whitchurch, have been similarly distinguished at various times. The fact proves the right of members of the corps to rank as officers in an army where they have gained such honours in the midst of military dangers. That members of the medical profession are singularly regardless of endangering their lives the death roll of the medical schools can prove. In the battlefield against disease, or in the midst of warfare, there have never been lacking fit candidates for the Victoria Cross from the ranks of medical men.

It is a healthy and welcome proof of interest in the voluntary hospitals that Londoners have again protested against the action of the Hospital Saturday Fund in continuing to hold street collections. The Council have earned unpopularity by not following the wise counsels given by the chairman of the Fund at the annual meeting in March. Street collections offer a premium to evil practices, which they indeed foster, and they further, and justly, irritate the public, who, we fear, do not understand that the hospitals and the police are equally powerless to prevent them so long as the Hospital Saturday Fund authorities persist in patronising them. The Hospital Saturday Fund is administered by worthy and enlightened men, and its powers for good are undeniably great. Why, therefore, it permits the continuance of a system which mars its usefulness and may actually endanger the existence of the Fund itself passes general comprehension. To our oft-repeated protest is this year added the ever-growing voice of Public Opinion, which is becoming unanimous in its judgment that street collections are not only a public nuisance, but exceedingly dangerous to public morals.

